# Persistent DNA damage response drives cGAS‐STING mediated neuroinflammation in a mouse model of tauopathy

**DOI:** 10.1002/alz.093509

**Published:** 2025-01-03

**Authors:** Himanshi Singh, Jianfeng Xiao, Sazzad Khan, Asmita Das, Tayebeh Pourmotabbed, Mohammad Moshahid Khan

**Affiliations:** ^1^ University of Tennessee Health Science Center, Memphis, TN USA; ^2^ Delhi Technological University, Memphis, TN USA

## Abstract

**Background:**

Tauopathies are a group of neurological disorders including Alzheimer’s disease that involve progressive neurodegeneration, behavioral deficits, and aberrant tau accumulation. While the molecular mechanisms that regulate the progression of the tauopathy are not fully elucidated, there is evidence to suggest that accumulation of nuclear DNA damage, particularly nuclear DNA double‐strand breaks (DNA DSBs), contribute to the progression of neurodegeneration. In our present work, we investigated the relationship between DNA DSB accumulation and neuroinflammation in the brains of AD patients and a mouse model of tauopathy.

**Method:**

We used multiple approaches including ELISA, RT‐qPCR, Western blot and immunohistochemistry to examine the DNA DSB expression and DNA repair function in the entorhinal cortex of human AD and non‐AD brains.

**Result:**

In comparison to non‐AD subjects, we found increased g‐H2A.X (Ser139) a biomarker of DNA DSB and reduced expression levels of DNA repair proteins in the entorhinal cortex of human AD brains. This was positively correlated with upregulation of cyclic GMP‐AMP synthase (cGAS) and stimulator of interferon genes (STING) immune regulatory pathway, and tau pathology. Next, we observed higher amounts of DNA DSB and altered expression levels of DNA repair proteins in the brains of PS19Tg mice as compared to non‐Tg mice. Our results showed that increased DNA DSBs and reduced expression of DNA repair proteins were further associated with significant increase in cGAS‐STING pathway.

**Conclusion:**

These results offered strong evidence that tauopathy is linked to DNA DSB accumulation and/or changes in DNA repair proteins, which may have an impact on a critical initial step of the process, such as neuroinflammation, leading to neurodegeneration and memory loss.

